# Supplementary material for: Targeting the Annexin A1-FPR2/ALX pathway for host-directed therapy in dengue disease
Source: eLife. 2022 Mar 16;11:e73853. doi: 10.7554/eLife.73853 (PMC8959599; doi:10.7554/eLife.73853)
Supplement: Supplementary file 1. — Patients were included in this study if DENV infection was confirmed by dengue specific IgM capture ELISA and/or real-time reverse transcriptase-polymerase chain reaction (RT-PCR). RT-PCR was conducted in RNA purified from human plasma using the primers and probes depicted in this table. [file elife-73853-supp1.docx]

**Supplementary Table 1: oligo primers and probes used in clinical samples.**

| **Serotype** | **Sequence of Primers and Probes (5’-3’)** |
| --- | --- |
| **DENV1-F** | ATCCATGCCCAYCACCAAT |
| **DENV1-R** | TGTGGGTTTTGTCCTCCATC |
| **DENV1-Probes** | **FAM**-TCAGTGTGGAATAGGGTTTGGATAGAGGAA-**TAMRA** |
| **DENV2-F** | TCCATACACGCCAAACATGAA |
| **DENV2-R** | GGGATTTCCTCCCATGATTCC |
| **DENV2- Probes** | **FAM**-AGGGTGTGGATTCGAGAAAACCCATGG-**TAMRA** |
| **DENV3-F** | TTTCTGCTCCCACCACTTTC |
| **DENV3-R** | CCATCCYGCTCCTTGAGA |
| **DENV3- Probes** | **TET**-AAGAAAGTTGGTAGTTCCCTGCAGACCCCA-**TAMRA** |
| **DENV4-F** | GYGTGGTGAAGCCYCTRGAT |
| **DENV4-R** | AGTGARCGGCCATCCTTCAT |
| **DENV4- Probes** | **TET**-ACTTCCCTCCTCTTYTTGAACGACATGGGA-**TAMRA** |
