# Supplementary material for: Targeting the Annexin A1-FPR2/ALX pathway for host-directed therapy in dengue disease
Source: eLife. 2022 Mar 16;11:e73853. doi: 10.7554/eLife.73853 (PMC8959599; doi:10.7554/eLife.73853)
Supplement: Transparent reporting form [file elife-73853-transrepform1.docx]

***eLife's* transparent reporting form**

We encourage authors to provide detailed information *within their submission* to facilitate the interpretation and replication of experiments. Authors can upload supporting documentation to indicate the use of appropriate reporting guidelines for health-related research (see [EQUATOR Network](http://www.equator-network.org/%20)), life science research (see the [BioSharing Information Resource](https://biosharing.org/)), or the [ARRIVE guidelines](http://www.plosbiology.org/article/info:doi/10.1371/journal.pbio.1000412) for reporting work involving animal research. Where applicable, authors should refer to any relevant reporting standards documents in this form.

If you have any questions, please consult our Journal Policies and/or contact us: [editorial@elifesciences.org](mailto:editorial@elifesciences.org).

**Sample-size estimation**

- You should state whether an appropriate sample size was computed when the study was being designed
- You should state the statistical method of sample size computation and any required assumptions
- If no explicit power analysis was used, you should describe how you decided what sample (replicate) size (number) to use

Please outline where this information can be found within the submission (e.g., sections or figure legends), or explain why this information doesn't apply to your submission:

The sample size estimation was done using G*Power 3.1 Software (Jacob Cohen. Psychological Bulletin Journal, 1992). This information can be found in the Statistics section of the manuscript. Sample size estimation was calculated considering the mean and standard deviation of hematocrit levels in DENV- and mock-infected animals (St John et al. 2013. DOI: 10.7554/eLife.00481) and mean and standard deviation of articular inflammation in wild-type and Annexin A1 KO animals (Kao et al. 2014. DOI: 10.1111/bph.12768). This calculation is also required for the animal ethics committee approval.

**Replicates**

- You should report how often each experiment was performed
- You should include a definition of biological versus technical replication
- The data obtained should be provided and sufficient information should be provided to indicate the number of independent biological and/or technical replicates
- If you encountered any outliers, you should describe how these were handled
- Criteria for exclusion/inclusion of data should be clearly stated
- High-throughput sequence data should be uploaded before submission, with a private link for reviewers provided (these are available from both GEO and ArrayExpress)

Please outline where this information can be found within the submission (e.g., sections or figure legends), or explain why this information doesn't apply to your submission:

A biological replication means that we performed the same test with biologically distinct samples, while a technical replicate implies that the same sample was assayed more than once. All the information about sample size and biological replicates for each experiment can be found in the figure legends.

In our work, all experiments were repeated at least twice, and the figures show the result of one representative experiment. Information regarding how often each experiment was performed can be found in the methodology section of the manuscript.

For the human volunteers, criteria for exclusion/inclusion of data are clearly stated in the methodology section of the manuscript.

**Statistical reporting**

- Statistical analysis methods should be described and justified
- Raw data should be presented in figures whenever informative to do so (typically when N per group is less than 10)
- For each experiment, you should identify the statistical tests used, exact values of N, definitions of center, methods of multiple test correction, and dispersion and precision measures (e.g., mean, median, SD, SEM, confidence intervals; and, for the major substantive results, a measure of effect size (e.g., Pearson's r, Cohen's d)
- Report exact p-values wherever possible alongside the summary statistics and 95% confidence intervals. These should be reported for all key questions and not only when the p-value is less than 0.05.

Please outline where this information can be found within the submission (e.g., sections or figure legends), or explain why this information doesn't apply to your submission:

All data were presented as the mean ± SD. GraphPad Prism 9.1.2 was used to determine statistical significance. Statistical significance was set as p ˂ 0.05. This information can be found in the methodology section of the manuscript. All p values are indicated in each figure legend. The figure legend also depicts the statistical test used, and the posthoc test applied.

(For large datasets, or papers with a very large number of statistical tests, you may upload a single table file with tests, Ns, etc., with reference to sections in the manuscript.)

**Group allocation**

- Indicate how samples were allocated into experimental groups (in the case of clinical studies, please specify allocation to treatment method); if randomization was used, please also state if restricted randomization was applied
- Indicate if masking was used during group allocation, data collection and/or data analysis

Please outline where this information can be found within the submission (e.g., sections or figure legends), or explain why this information doesn't apply to your submission:

Mice were randomly allocated into experimental groups using an MS Excel randomization tool. This information can be found in the methodology section.

**Additional data files ("source data")**

- We encourage you to upload relevant additional data files, such as numerical data that are represented as a graph in a figure, or as a summary table
- Where provided, these should be in the most useful format, and they can be uploaded as "Source data" files linked to a main figure or table
- Include model definition files including the full list of parameters used
- Include code used for data analysis (e.g., R, MatLab)
- Avoid stating that data files are "available upon request"

Please indicate the figures or tables for which source data files have been provided:

The numerical data that are represented in figures 1-6 are provided as source data.
